# Associations of Accelerometer‐Measured Sedentary Behavior and Gray Matter Volume in Healthy Young Adults

**DOI:** 10.1002/ejsc.12310

**Published:** 2025-04-28

**Authors:** Marco Giurgiu, Anastasia Benedyk, Markus Reichert, Oksana Berhe, Urs Braun, Ulrich Ebner‐Priemer, Heike Tost, Andreas Meyer‐Lindenberg

**Affiliations:** ^1^ Department of Psychiatry and Psychotherapy Central Institute of Mental Health, Medical Faculty Mannheim/Heidelberg University Mannheim Germany; ^2^ Mental mHealth Lab Institute of Sports and Sports Science Karlsruhe Institute of Technology Karlsruhe Germany; ^3^ Department of eHealth and Sports Analytics Ruhr‐University Bochum Bochum Germany

**Keywords:** brain health, gray matter, MRI, physical behavior, wearable, young adults

## Abstract

Sedentary lifestyles can be seen as one of the central risk factors for poor health in the 21st century. Previous studies indicated negative associations between sedentary behavior and brain health. However, the neurological link between sedentary behavior and gray matter volume remains unclear. This study aimed to assess the relationship between device‐based measured sedentary time and gray matter volume in healthy young adults. A total of 181 participants wore a move‐II hip‐accelerometer to measure sedentary time and physical activity over seven consecutive days. Following the study week, participants underwent a structural magnetic resonance imaging (MRI) scan to assess gray matter volume. Whole‐brain voxel‐based morphometry analysis was conducted on the MRI data, and group comparisons focused on a region of interest to examine the potential association of moderate‐to‐vigorous intensity physical activity (MVPA). On a daily average, participants spent 6.04 h (SD = 2.2) in sedentary and 1.2 h (SD = 0.51) in MVPA. More sedentary time was associated with lower gray matter volume in the left superior frontal gyrus (pFWE = 0.007). Furthermore, participants with high levels of MVPA demonstrated higher gray matter volume in the left (pFWE = 0.028) and right (pFWE = 0.022) superior frontal gyrus compared to the sedentary group with low MVPA engagement. Sedentary behavior may be linked to smaller gray matter volume in brain structure, particularly in the superior frontal gyrus, which plays an important role in motor and cognitive brain networks. Intriguingly, people with high sedentary behavior but concurrently high levels of MVPA did not exhibit this negative gray matter association.

**Summary**

- Previous research has shown a positive association between physical activity and gray matter volume in various brain regions. Conversely, sedentary time has been associated with negative effects on brain health, such as reduced motor competence and potential impairments in executive function.
- The association of accelerometer‐measured sedentary time and brain structure revealed a significant reduction of gray matter volume in the superior frontal gyrus among healthy young adults but not in people with high MVPA engagement. Thus, high levels of MVPA may attenuate the negative effects of sedentary time on brain health.
- Independent of overall sedentary time, physical activity with higher intensities might be beneficial for young adults’ brain health. This study calls for experimental validation of its observational findings and underlines an urgent need for interventions that promote physical activity to mitigate the negative effects of sedentary behavior on brain health.

## 1 Introduction

Acknowledged by the latest WHO Guidelines, a sedentary lifestyle can be seen as one of the central risk factors for health in the 21st century (World Health Organization 2020). Nowadays, adolescents spent around 8.2 h and adults around 6.5 h in a sedentary position across different areas of life such as at work/school, during transportation, or leisure time (Gardner et al. 2019; Yang et al. 2019). Sedentary behavior (SB) refers to any waking activity characterized by an energy expenditure of 1.5 metabolic equivalents or less while in a sitting, reclining, or lying posture (Tremblay et al. 2017). Empirical evidence reveals that prolonged sedentary time is associated with various negative health outcomes, including cardiometabolic disease, obesity, all‐cause mortality, brain functions, or depression (Kandola et al. 2021; Saunders et al. 2020; Silveira et al. 2022). Although daily physical activity (PA; defined as any bodily movement produced by skeletal muscles that results in energy expenditure) [(Caspersen et al. 1985) > 1.5 MET] and higher intensities, such as moderate‐to‐vigorous physical activity (MVPA), may attenuate the health risks of sedentary time (Ekelund et al. 2020; Zou et al. 2024); there is a worldwide trend that adolescents, as well as adults, are not sufficiently active (Guthold et al. 2020). The physiology of SB reveals various negative health consequences (Pinto et al. 2023), whereas in contrast, the impact on brain health remains understudied (Zou et al. 2024).

In recent years, there has been growing interest in the potential link between SB and brain health [e.g., the impact on specific cognitive functions such as working memory, attention, or processing speed (Zou et al. 2024)]. For example, Chandrasekaran and colleagues postulate hypotheses and discuss several physiological mechanisms including but not limited to changes in brain structure [e.g., gray matter volume) that can explain the negative effects of SB on cognitive functions (Chandrasekaran et al. 2021]. A systematic review based on eight studies reported that higher levels of SB is associated with lower cognitive performance. In particular, Falck et al. (2017) concluded that limiting sedentary time and engaging in regular MVPA may best promote healthy cognitive aging among adults ≥ 40 years (Falck et al. 2017). Similar results were found by Choudhary and colleagues while presenting a summary of studies that reported a cognitive decline related to sedentary activities (Choudhary et al. 2021). Pindus et al. (2021) showed that prolonged SB was related to greater interference in an inhibitory control trial among adults (Pindus et al. 2021). A further systematic review based on 45 articles concluded that higher PA and lower SB are associated with better global cognitive function in older adults (Rojer et al. 2021). In general, a considerable amount of literature has been published on the impact of PA on cognitive functions. Some studies indicated that PA, especially at higher intensities, can preserve or improve cognition in an acute (Chang et al. 2012) and chronic manner (Hoffmann et al. 2021; Sewell et al. 2023). Contrary, some studies reported no association between PA and cognition (Carter et al. 2020; Gilmour et al. 2023) as well as interventions that intend to reduce SB (Magnon et al. 2018).

Although several putative physiological mechanisms have been proposed in the literature (Chandrasekaran et al. 2021) to explain the detrimental effect of higher levels of SB on cognitive performance (i.e., changes in gray matter volume), the number of empirical studies that focused on the link between SB and brain structure in younger adults is very limited (Zou et al. 2024). For example, Paruk et al. (2020) conducted brain volumetric analyses while comparing sedentary persons and ultraendurance athletes. The authors showed that athletes have increased gray, white, and total matter volume but on a regional analysis level smaller regional gray matter (GM) volume (Paruk et al. 2020). In a sample of younger adults, Pindus et al. (2020) concluded based on their findings that SB may reduce neurofunctional readiness for top–down control, whereas MVPA was not related. In a sample of overweight children, Migueles et al. (2020) indicated that reducing SB and increasing MVPA are associated with greater GM volume in the right hippocampus. Further studies in the context of SB and brain health focused on the association between SB, PA, and brain atrophy. For example, a previous study found that adults aged ≥ 50 years with diagnosis of knee osteoarthritis who engage in more MVPA, regardless of SB, exhibited greater cortical thickness in regions, such as left superior frontal gyrus and temporal pole, which are susceptible to age‐associated atrophy (Falck et al. 2020). Furthermore, Arnardottir and colleagues demonstrated a correlation between device‐based measurements of SB and PA with brain atrophy among older adults (Arnardottir et al. 2016). These first findings reveal a potential link between SB and lower GM volume but come again with some methodological shortcomings. Although the abovementioned studies provide initial evidence documenting a potential link between higher levels of SB and lower GM volume, they assessed SB via self‐reported questionnaires, which are prone to retrospective underestimation (Prince et al. 2020), especially because SB can be seen as an invisible behavior with a less accessible cognitive representation (Gardner et al. 2019). This is especially critical since the assessment of SB is more prone to recall biases compared to the surveying of PA. Buttressing the findings of studies using self‐reported SB measures by those assessing SB via devices is important because Prince and colleagues observed that self‐reports underestimated sedentary time by around 1.74 h/day compared to device measures (Prince et al. 2020).

Based on preliminary findings (Arnardottir et al. 2016; Falck et al. 2020; Migueles et al. 2017; Zavala‐Crichton et al. 2020) and from a preventive perspective the urgent issue to understand the link between SB and brain structure in younger adults (Zou et al. 2024), we analyzed data from a cross‐sectional study utilizing a multimodal approach combining device‐based assessed SB and PA via accelerometers and MRI measures in a community‐based sample. First, we conducted a whole‐brain analysis to test the association between SB and GM volume. Second, given the extensive evidence linking PA to GM volume across a broad range of brain regions (Batouli and Saba 2017), and the ongoing discussion surrounding the potential of PA levels to mitigate negative health effects of SB (Biswas et al. 2015; Ekelund et al. 2016; Stamatakis et al. 2019), we researched a potential influence of MVPA (Stamatakis et al. 2018). To this end, this study aimed to investigate potential group differences in gray matter volume between individuals with different PA and SB profiles. In particular, based on the classification approach provided by Pinto et al. (2023), this study compared younger adults who were highly sedentary but differed in their PA levels (i.e., spent more or less time in MVPA).

## 2 Methods

### 2.1 Participants

Between December 2014 and September 2019, 351 participants were recruited from population registers using a two‐stage proportionally layered procedure, which was stratified by age, sex, and nationality. The current study used the data from 181 healthy young adults aged 18 to 28 who participated in both accelerometry assessment and structural MRI scan. The sample size was determined by resource limitations and feasibility considerations (Lakens 2022). For example, limited MRI scan time and limited number of sensors. Inclusion criteria for selection were young adults residing in the study region, specifically the Rhine–Neckar district. Participants were excluded if they reported any chronic endocrine, cardiovascular, immunological, or clinically manifested mental disorders. Participants with acute diseases or moderate to difficult impairment of intelligence and participants of consent or legal incapacity were excluded. The final sample consisted of 181 participants (48% females) with a mean age of 23.2 years (SD = 2.7) and a mean body mass index (BMI) of 23.2 kg/m^2^ (SD = 3.6; for detailed participant characteristics refer to Table 1).

**TABLE 1:** Participant characteristics (*N* = 181).

	Min	Max	Mean	SD
Age (years)	18.00	28.00	23.16	2.70
Sex			87 f/94 m	
Body mass index (kg/m^2^)	16.98	39.07	23.20	3.55
Total intracranial volume (cm^3^)	1253.37	1994.88	1614.98	138.69
Employment status (employed/unemployed)			75/106	
Smoking (nonsmoker/smoker)			139/42	
Socioeconomic status (25)	5.50	20.80	14.24	3.28
Accelerometer wear time (h)	6	20.23	12.52	2.53
Movement acceleration (mg)	19.36	144.91	68.55	21.44
Sedentary time (h)	1.70	14.92	7.02	2.23
Moderate‐to‐vigorous physical activity time (h)	0.07	3.19	1.2	0.51

The study was conducted in accordance with the ethical guidelines for medical research as specified by the Declaration of Helsinki. The research protocol was approved. Prior to enrollment in the study, all eligible participants received information about the study procedures and provided written informed consent. Participants were also informed of their right to withdraw from the study at any time. Monetary compensation was provided to all participants for their study participation. No surrogate consent procedure was employed in this study.

### 2.2 Study Procedures

Participants worn an accelerometer (movisens Move II or III, movisens GmbH, Karlsruhe, Baden‐Wuerttemberg, Germany, www.movisens.com) for seven consecutive days during their everyday lives. Before the assessment, participants received an extensive briefing regarding the usage the accelerometer. After the 7‐day assessment period [i.e., represents a valid week for habitual movement behavior (Migueles et al. 2017)], participants returned the accelerometer and underwent structural magnetic resonance imaging (MRI) scans.

### 2.3 Accelerometry

Participants wore the accelerometer on the right side of their hip during the entire study week but not during sleep. Validation studies revealed the move accelerometer to be appropriate in assessing both human PA and SB (Anastasopoulou et al. 2014; Giurgiu et al. 2020). The triaxial acceleration sensor captured movements with a sampling frequency of 64 Hz and a resolution of 12 bits. The raw acceleration intensity was stored on an internal memory card. The calculation of both energy expenditure and MET occurs in two steps. First, an activity class (e.g., lying, sitting, walking, and cycling) is determined based on acceleration and barometric signals. Once the activity class is identified, the corresponding model uses the acceleration metric movement acceleration, altitude changes derived from barometric data, and personal parameters, such as age, gender, weight, and height, to compute the energy expenditure or MET value (movisens Ltd 2022). We operationalized SB as time spent in a sitting/lying posture with low energy expenditure (≤ 1.5 MET) across the study week. Time spent in MVPA was operationalized as time spent in higher intensities (≥ 3.0 MET). For this purpose, raw acceleration data were processed in 1 min intervals using the software DataAnalyzer, version 1.6.12129 (movisens GmbH, Karlsruhe, Baden‐Wuerttemberg, Germany, www.movisens.com). Raw acceleration represents the vector magnitude of the acceleration in milli‐g assessed with the three sensor axes. This parameter is computed using a high‐pass filter (0.25 Hz) eliminating gravitational components and a low‐pass filter (11 Hz) excluding artifacts, such as vibrations when cycling on a rough road surface or shocks of the sensor.

### 2.4 Magnetic Resonance Imaging

Magnetic resonance imaging (MRI) was used to obtain structural brain scans on a 3‐T whole‐body Siemens Magnetom Trio scanner at the CIMH. T1‐weighted three‐dimensional magnetization‐prepared rapid acquisition gradient‐echo (MPRAGE) sequence was used to acquire structural scans with whole‐brain coverage, a spatial resolution of 1 mm^3^, repetition time = 2300 ms, echo time = 3.03 ms, inversion time = 900 ms, flip angle = 9°, 192 contiguous sagittal slices, 1 mm slice thickness, and a field of view of 256 mm. Voxel‐based morphometry (VBM), an automated whole‐brain processing method, was utilized to examine volumetric differences associated with prolonged SB. VBM analysis was conducted following the standard pipeline using the CAT12 toolbox (r934; Gaser et al. 2024) implemented in the statistical parametric mapping software (SPM12; https://www.fil.ion.ucl.ac.uk/spm/) executed in MATLAB 2017. Gray matter image processing involved standard procedures, including tissue classification, segmentation into gray matter, white matter, cerebrospinal fluid, and noncerebral tissue classes, and normalization to montreal neurological institute (MNI) space using a diffeomorphic image registration algorithm (DARTEL). Additional steps included correction for image intensity nonuniformity, cleaning up of gray matter partitions, application of a hidden Markov random field model, and spatial adaptive nonlocal means denoising. The resulting tissue segments were transformed into volume equivalents by multiplying the Jacobian determinants of the deformation field to the gray matter density values. The total intracranial volume (TIV) was computed for each participant and added as a covariate to the analyses. The segmented, normalized, noise‐corrected, and modulated gray matter images were smoothed with an 8 mm full width at a half maximum isotropic Gaussian kernel. Subsequently, images were visually inspected for scanner artifacts and structural abnormalities.

### 2.5 Statistical Analyses

**FIGURE 1:**
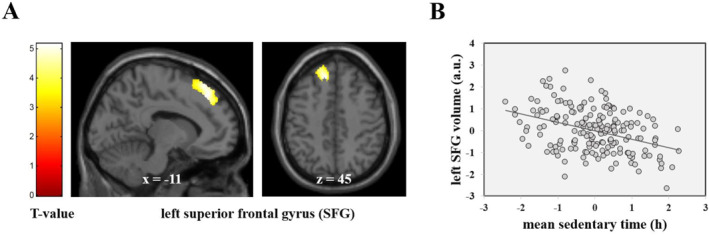
The total amount of sedentary time within the study week relates to gray matter volume of the super frontal gyrus (SFG). (A) T‐map of the significant negative association between mean sedentary time across the study week and gray matter volume in healthy young adults. The indicated cluster (*k* = 216) is whole‐brain significant at *p*_FWE_ < 0.05 level, highlighting a reduction in gray matter volume within the left superior frontal gyrus (SFG) region associated with prolonged SB. For illustration purposes, the finding is shown at a significance threshold of *p* < 0.001, uncorrected. (B) Scatterplot of the negative association between mean sedentary time across the study week (*x* axis; group‐centered SB) and left superior frontal gyrus GM volume [*y* axis; individual GM volume values for the peak voxel of the displayed cluster in (A), both residualized for age, gender, total intracranial volume (TIV), body mass index (BMI), and mean wear time, and employment status]. a.u., arbitrary units.

Individual preprocessed gray matter volume maps were analyzed in SPM12 using a mass univariate general linear model. To test whether the total amount of sedentary time within the study week related to the gray matter volume, we computed a general linear model with mean sedentary time as regressor of interest and age, gender, total intracranial volume, mean wear time of the accelerometer, employment status, and BMI as covariates. On the basis of our main finding, we conducted an ROI analysis in an a priori defined mask (16,127 voxels) comprising the left and right superior frontal gyrus (Jenkinson et al. 2012). For the ROI analysis, we used the small volume correction approach implemented in SPM12. The significance threshold for the ROI analysis was set to *p* < 0.05 family wise error (FWE) corrected for multiple comparisons across all voxels within the mask. Outside this prehypothesized ROI, findings were considered significant if they passed a significance threshold of *p* < 0.05 FWE corrected for multiple comparisons across the whole brain. Sensitivity analyses were conducted by including only days with at least 10 h accelerometer wear time (Troiano et al. 2008). In addition, we conducted a correlational analysis to further characterize the association between sedentary time and superior frontal gyrus gray matter volume. This analysis was performed using Pearson's correlation coefficient (*r*), with both variables residualized for age, gender, TIV, BMI, and nonwear time (Figure 1B). This analysis was performed using Pearson's correlation coefficient (*r*) in SPSS (ver. 28.0.0.0), with both variables residualized for age, gender, TIV, BMI, and nonwear time. We found a negative correlation (*r* = −0.38), which, according to commonly used thresholds, represents a moderate effect size (Cohen 1988).

To investigate the potential influence of MVPA on the brain in individuals who engage in higher sedentary time, we conducted an additional analysis comparing two groups. According to previous procedures 5, the selection of these groups was based on dividing the total time spent sedentary and engaging in MVPA into equally sized tertiles (low, median, and high), which were then combined. This approach resulted in the identification of two distinct groups of interest: (1) individuals with high levels of SB and low levels of MVPA and (2) individuals with high levels of SB and high levels of MVPA. Based on the results of the whole‐brain analysis, a bilateral mask of the significant cluster region (superior frontal gyrus as defined by the Harvard‐Oxford atlas 24) was used for further region‐of‐interest (ROI) analysis.

## 3 Results

### 3.1 Descriptive Statistics

**FIGURE 2:**
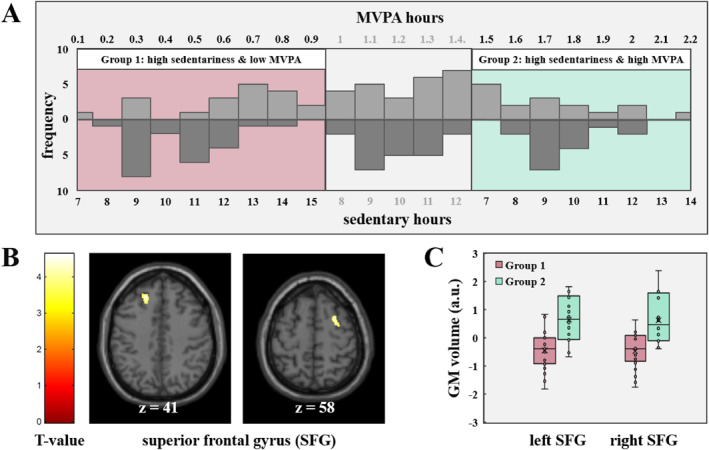
Comparison between high sedentary participants engaging in low and high levels of MVPA. (A) Group selection of participants who are highly sedentary (> 7.8 h of sitting per day) and show either rare (Group 1) or frequent (Group 2) MVPA engagement. The upper panel shows the distribution of MVPA hours divided into three equal‐sized tertiles, whereas the lower panel shows the distribution of sedentary hours in the respective groups. (B) T‐map of the significant comparison between high sedentary participants who engaged in low and high amounts of MVPA. The illustrated clusters are significant at the FWE‐corrected *p* < 0.05 level, small volume corrected by a mask of the bilateral superior frontal gyrus [as defined by the Harvard‐Oxford atlas (Jenkinson et al., 2012)]. High sedentary participants who engaged in more MVPA showed higher gray matter volume in the left and right superior frontal gyrus. (C) Boxplots showing the difference in GM volume in the left SFG and right SFG between groups (low vs. high levels of MVPA in high sedentary participants), residualized for age, gender, total intracranial volume (TIV), body mass index (BMI), and mean wear time and employment status.

On average, accelerometers were worn during waking time for 13.76 h per day and participant across the whole study week. The parameterization of the raw acceleration signals revealed that participants spent on average 6.04 h (SD = 2.3) in sedentary time and 1.3 h (SD = 0.55) in MVPA (see Table 1). The tertiles of the SB distribution were 4.9 h (33.33%) and 6.5 h (66.67%) and 1.05 h (33.33%) and 1.52 (66.67%) for the MVPA distribution. The combination of time spent sedentary and engaging in MVPA, resulted in two distinct groups of interest: (1) individuals with high levels of sedentary time and low levels of MVPA (*n* = 23) and (2) individuals with high levels of sedentary time and high levels of MVPA (*n* = 16; see Figure 2A).

### 3.2 Sedentary Time and Gray Matter Volume

Consistent with our initial expectation, the whole‐brain VBM analysis revealed a significant negative association between the daily average amount of participants’ sedentary time during the study week and gray matter volume. In particular, participants with higher levels of SB displayed less GM volume in the left dorsolateral superior frontal gyrus (SFG) [T (173) = 5.15; pFWE = 0.007; and Table 2, analysis I] when compared to their less sedentary counterparts (refer to Figure 1). No other regions of the brain demonstrated a significant effect at the whole‐brain FWE‐corrected level. We additionally conducted a sensitivity analysis by keeping only days with more than 10 h wear time and found less GM in the same brain region [T (159) = 5.04, pFWE = 0.012; and Table 2, analysis II].

**TABLE 2:** Brain regions showing negative association with SB and group comparison of high sedentary participants engaging in high versus low MVPA levels.

Contrast	Brain region	Peak voxel			*k*	*T*	pFWE_corr_
		*x*	*y*	*z*			
Analysis I							
Neg. association	Left superior frontal gyrus	−10	34	45	216	5.15	0.007*
Analysis II							
Neg. association	Left superior frontal gyrus	−10	38	52	118	5.04	0.012*
Analysis III							
High MVPA > low MVPA	Left superior frontal gyrus	−18	34	42	65	4.53	0.028*
	Right superior frontal gyrus	27	8	60	87	4.62	0.022*

*Note:* Coordinates represent the center of each significant cluster in standardized MNI space with a right‐handed coordinate system and the center of the anterior commissure as the origin. Cluster extent *k* is given a pFWE < 0.05, family wise error corrected for multiple comparisons whole‐brain (analyses I and II) and within the relevant regions of interest using small volume correction (analysis III).

### 3.3 Group Comparison

Given the observed reduction in GM volume within the SFG in highly sedentary participants, we expected a significant difference between the selected groups in this particular brain region. Our ROI analysis within the SFG revealed a significant difference between the two groups with high levels of SB but differing levels of MVPA engagement during the study week. Specifically, when compared to the sedentary group with low MVPA engagement, participants with high levels of MVPA engagement demonstrated higher gray matter volume in the left [T (31) = 4.53, pFWE = 0.028; corrected within ROI] and right [T (31) = 4.62, pFWE = 0.022; corrected within ROI; Table 2] superior frontal gyrus. Significant clusters indicating group differences between the highly and low physically active participants who spend high amounts of time while sitting are presented in Figure 2 and Table 2 analysis III.

## 4 Discussion

In this study, we found a robust negative association between sedentary time and GM volume in the left superior frontal gyrus, specifically in its dorsolateral portion bordering on the supplementary motor area (SMA). Our data further show that participants with high levels of MVPA had higher gray matter volume in the left and right SFG when compared to sedentary participants who did not engage in MVPA to the same extent. Previous studies indicated that MVPA may attenuate the negative effects of SB on physiological health parameters (Ekelund et al. 2020), which makes us tempting to speculate that high loads of MVPA may reduce the potential negative effects of SB on brain health. For example, in a sample of indviduals with early osteoarthritis (aged of at least 50 years) Falck et al. (2020) have shown that higher MVPA was associated with greater cortical thickness in the temporal pole and superior frontal gyrus of the left hemisphere independent of SB (Falck et al. 2020).

Taking a neurobiological perspective, the SFG is known to be composed of multiple subregions that are anatomically and functionally involved in various networks including the motor, cognitive control, and default mode networks (W. Li et al. 2013). The posterior part of the SFG, which includes the SMA, is mainly activated during motor tasks and plays an important role in the control of complex motor functions and coordinated hand movements (Chouinard and Paus 2010; Martino et al. 2011). For example, Adank et al. (2018) study found a negative association between motor competence and SB and a positive association between motor competence and MVPA among 7–11 years old children. Hardy and colleagues identified that each hour of screen time was associated with lower odds of achieving locomotor skills in a sample of children aged 5–16 years (Hardy et al. 2018). In line with those studies, Lopes et al. (2012) suggested that PA levels may not overcome the deleterious influence of high levels of SB on motor coordination. Although the studies provide first insights into the association between SB and motor competence among children, none of these studies focused on younger adults. This underscores a significant gap in the literature, as most studies on younger adults have focused on the impact of PA or fitness on specific aspects of brain health, whereas evidence linking SB to brain health in this age group is relatively scant (Zou et al. 2024).

Moreover, the dorsolateral part of the SFG is involved in executing functions (Boisgueheneuc et al. 2006; Owen 2000) and attention processing (de Borst et al. 2012; Corbetta and Shulman 2002). Of note, the left SFG was found to be particularly important for spatially oriented processing and working memory, which are both critical aspects of executive functioning (Boisgueheneuc et al. 2006). In detail, executive functions are essential for daily activities such as decision‐making, problem‐solving, and planning. Previous studies addressing acute and chronic association between SB and executive functions revealed mixed evidence. Thus, our observed association between SB and reduced SFG gray matter volume may have implications for these cognitive functions. For example, Li and colleagues have shown that acute effects of interrupting prolonged sitting neither improved nor impaired cognitive performance (J. Li et al. 2022). Although focusing on chronic effects, similar results were found by Cox et al. showing cognitive function to remain within the normal range regardless of PA or sitting time (Peng Cox et al. 2021). In contrast, Wanders et al. (2021) found a strong independent positive association between chronic total SB and cognitive function in a heterogeneous population. Although this relation was not consistent across different domains, their study highlighted that especially work‐ and computer‐related SB was positively associated with cognitive function, whereas Chandrasekaran et al. (2024) observed no associations between device‐measured SB and executive functions. Hoang et al. (2016) investigated the association between 25‐year patterns of television viewing as a maker of SB and PA and midlife cognition. The authors concluded that high television viewing and low PA in early adulthood were associated with worse midlife executive function and processing speed (Hoang et al. 2016). In line with our findings, Magnon and colleagues reported that past SB only negatively predicted cognitive inhibition when the PA level was low (Magnon et al. 2021). Similar effects were identified by Rojer and colleagues (Rojer et al. 2021). In particular, higher PA and lower SB were associated with better global cognitive function in older adults, suggesting that greater duration of high‐intensity PA could be most beneficial for global cognitive function. Future studies may also account for the influence of LPA, which is often neglected in scientific practice and recommendations (Ross et al. 2024) but can also exert a positive effect on cognition (Erlenbach et al. 2021).

In contrast to the research on SB and brain morphology, the effects of PA have been studied in more detail. For example, a cross‐sectional study have shown that PA dose and intensity were independently associated with larger brain volumes, gray matter density, and cortical thickness of several brain regions (Fox et al. 2022). A further cross‐sectional shows that PA at work time changed functional connectivity between a location in the middle/posterior hippocampus and regions of the default mode network and between a location in the anterior hippocampus and regions of the somatomotor network (Seoane et al. 2022). In line with these findings, Cherednichenko et al. (2024) study concluded that intense PA, such as MVPA, may contribute to enhancing the hippocampal volume in young adults (Cherednichenko et al. 2024). In a sample among healthy monozygotic twin pairs in their mid‐30s, Rottensteiner et al. (2015) have shown that greater level of PA (i.e., a difference of ≥ 1.5 MET per hour per day) is associated with improved prefrontal cortex gray matter volume independent of genetic background (Rottensteiner et al. 2015).

The physiological mechanism of action remains thus far elusive. Chandrasekaran and colleagues (Chandrasekaran et al. 2021) postulate some hypotheses of plausible mechanisms for the putative effects of breaking prolonged sitting on improving cognitive function. A recent published overview by Zou et al. (2024) discussed that potential mechanisms might be moderated by the level of mental activation in terms of differentiating between being mentally passive (e.g., watching TV) or being mentally active (e.g., reading). Future research could tackle this issue by assessing the level of mental activation through ambulatory assessment, such as self‐reports via smartphones, in the daily lives of younger adults (Reichert, Braun, et al. 2020). Moreover, further research is needed to replicate our findings and to tackle the hypothesis that a pronounced sedentary lifestyle without sufficient MVPA may impair executive functions, such as reducing working memory capacity. One approach might be to combine MRI and accelerometer data with real‐time cognitive assessments on smartphones to detect associations between SB and working memory or inhibition control. Sliwinski et al. demonstrate that brief cognitive assessments made in uncontrolled naturalistic settings provide measurements that are comparable in reliability to assessments made in controlled laboratory environments (Sliwinski et al., 2018).

Some limitations of our work merit further discussion. First, we assessed SB via accelerometry by using a hip‐worn location over 1 week, which varies from the recommendation of using thigh‐worn devices (Stevens et al., 2020). However, a previous validation study indicates that using the move sensor at the hip accurately detects SB and covers both aspects of SB (i.e., postural and intensity information) (Giurgiu et al., 2020). Second, our additional group comparison might be limited by the number of participants for each group. Future studies may replicate our findings in different target groups with higher sample sizes. Third, our findings cannot be generalized to other age groups. However, our results were stable across a community‐based sample of young adults. Fourth, our naturalistic data do not prove causality (Susser, 1991). Moreover, our results do not offer insights into a potential reverse association, such as whether device‐measured SB later in life is linked to current or past cross‐sectional measures of brain atrophy (Arnardottir et al., 2016). Future research endeavors might address causality in real‐time intensive longitudinal studies by applying experimental approaches such as manipulating behavior through just‐in‐time‐adaptive interventions [i.e., providing the right type/amount of support, at the right time, and by adapting to an individual’s changing internal and contextual state (Nahum‐Shani et al., 2018)]. For example, encouraging individuals to replace sedentary time with **PA** based on their personal behavior within their living environment, while delivering digital prompts via smartphone (Bronas et al., 2024). Fifth, studies have shown that sleep and light physical activity (LPA) are also related to executive functions (Anderson et al., 2009; Zhu et al., 2022), whereas another study highlighted no significant differences in executive functions between participants achieving the MVPA guidelines and those that did not (Gilmour et al., 2023). Future studies might be interested in dissolving the optimum composition within a 24 h cycle of spending time in sleep, SB, LPA, and MVPA for enhancing executive functioning by applying compositional data analysis.

## 5 Conclusions

In conclusion, our study provides evidence that device‐measured SB in daily life is associated with reduced GM volume, particularly in the left superior frontal gyrus, which plays an important role in motor and cognitive functions. However, engaging in high levels of MVPA may attenuate negative effects on GM volume. Future studies should further investigate the impact of SB and MVPA on brain structure and function using both morphometric and functional neuroimaging techniques combined with device‐based assessment of physical behavior with a particular focus on experimental designs that allow for more causal conclusions. This knowledge may provide insights into potential benefits of **PA** in reducing the negative effects of SB on brain health.

## Author Contributions

Study concept and management: MR, UE‐P, HT, AM‐L. Data analysis: MG, AB, MR. Data review and interpretation, and manuscript writing: MG and AB. All authors then provided feedback on consequent drafts until an agreement was reached on the published version of the manuscript. MG and AB, the guarantors, accepts full responsibility for the work and conduct of the study, had access to the data, and controlled the decision to publish.

## Conflicts of Interest

A.M.‐L. has received consultant fees from Daimler und Benz Stiftung, EPFL Brain Mind Institute, Fondation FondaMental, Hector Stiftung II, Invisio, Janssen‐Cilag GmbH, Lundbeck A/S, Lundbeckfonden, Lundbeck Int. Neuroscience Foundation, Neurotorium, MedinCell, The LOOP Zürich, University Medical Center Utrecht, University of Washington, Verein für Mentales Wohlbefinden, von Behring‐Röntgen‐Stiftung; speaker fees from Ärztekammer Nordrhein, Caritas, Clarivate, Dt. Gesellschaft für Neurowissenschaftliche Begutachtung, Gentner Verlag, Landesärztekammer Baden‐Württemberg, LWL Bochum, Northwell Health, Ruhr University Bochum, Penn State University, Society of Biological Psychiatry, University Prague, Vitos Klinik Rheingau; and editorial and/or author fees from American Association for the Advancement of Science, ECNP, Servier Int., Thieme Verlag.

U.E.‐P. received fees for consulting from Boehringer‐Ingelheim and speaker honorarium from Angelini Pharma, which is not related to the submitted work.

The other authors report no biomedical financial interests or potential conflicts of interest.
